# Hypertension and Prohypertensive Antineoplastic Therapies in Cancer Patients

**DOI:** 10.1161/CIRCRESAHA.121.318051

**Published:** 2021-04-02

**Authors:** Daan C.H. van Dorst, Stephen J.H. Dobbin, Karla B. Neves, Joerg Herrmann, Sandra M. Herrmann, Jorie Versmissen, Ron H.J. Mathijssen, A.H. Jan Danser, Ninian N. Lang

**Affiliations:** 1 Division of Vascular Medicine and Pharmacology, Department of Internal Medicine (D.C.H.v.D., J.V., A.H.J.D.), Erasmus MC University Medical Center, Rotterdam, The Netherlands.; 2 Department of Medical Oncology, Erasmus MC Cancer Institute (D.C.H.v.D., R.H.J.M.), Erasmus MC University Medical Center, Rotterdam, The Netherlands.; 3 Department of Hospital Pharmacy (J.V.), Erasmus MC University Medical Center, Rotterdam, The Netherlands.; 4 BHF Glasgow Cardiovascular Research Centre, Institute of Cardiovascular and Medical Sciences, University of Glasgow, United Kingdom (S.J.H.D., K.B.N., N.N.L.).; 5 Department of Cardiovascular Medicine (J.H.), Mayo Clinic, Rochester, MN.; 6 Division of Nephrology and Hypertension (S.M.H.), Mayo Clinic, Rochester, MN.

**Keywords:** angiogenesis inhibitors, antineoplastic agents, comorbidity, hypertension, neoplasms, prognosis, risk factors

## Abstract

The development of a wide range of novel antineoplastic therapies has improved the prognosis for patients with a wide range of malignancies, which has increased the number of cancer survivors substantially. Despite the oncological benefit, cancer survivors are exposed to short- and long-term adverse cardiovascular toxicities associated with anticancer therapies. Systemic hypertension, the most common comorbidity among cancer patients, is a major contributor to the increased risk for developing these adverse cardiovascular events. Cancer and hypertension have common risk factors, have overlapping pathophysiological mechanisms and hypertension may also be a risk factor for some tumor types. Many cancer therapies have prohypertensive effects. Although some of the mechanisms by which these antineoplastic agents lead to hypertension have been characterized, further preclinical and clinical studies are required to investigate the exact pathophysiology and the optimal management of hypertension associated with anticancer therapy. In this way, monitoring and management of hypertension before, during, and after cancer treatment can be improved to minimize cardiovascular risks. This is vital to optimize cardiovascular health in patients with cancer and survivors, and to ensure that advances in terms of cancer survivorship do not come at the expense of increased cardiovascular toxicities.

Over the last few decades, the development of novel anticancer therapies has markedly increased survival rates for patients with a wide variety of malignancies.^1^ In 2019, almost 17 million cancer survivors were alive in the United States alone, and this number is predicted to increase to >22 million by 2030.^2^ This improved survival comes at the cost of potential short- and long-term toxicities associated with anticancer drugs. Cardiovascular toxicities are prominent and adversely affect outcomes in cancer survivors.^3,4^ While the cardiovascular toxic effects of older conventional chemotherapeutic drugs, such as anthracyclines and antimetabolites, have received considerable attention, there is a growing awareness of the importance and detrimental vascular effects of newer generation anticancer agents, particularly targeted therapies.^5–8^ These adverse vascular sequelae are a major focus of scientific and clinical endeavor in cardio-oncology, a rapidly growing subspeciality that aims to optimize cardiovascular care and health for patients with cancer.^9,10^

Systemic hypertension is one of the most frequently encountered vascular toxicities of many anticancer therapies and is a major risk factor for cardiovascular disease (CVD), including heart failure, stroke, myocardial infarction, and cardiac arrythmias,^11^ as well as renal disease.^12^ Over the years, a better insight into the diverse mechanisms by which antineoplastic agents induce hypertension has been obtained, but gaps in our understanding remain. Of note, some anticancer therapies cause an acute rise in blood pressure, which may result in deterioration of preexisting cardiovascular conditions and lead to acute hypertension-related complications in severe cases.^13,14^ Consequently, these hypertension-induced complications might require a reduction of treatment dosage or even discontinuation from potentially life-saving anticancer treatment, impairing patient survival. Distinct from the development of rapid-onset hypertension, several antineoplastic agents are associated with hypertension many years after the initial treatment period. This is reflected by an increased prevalence of hypertension in long-term survivors of both childhood and adult-onset cancers compared with the general population. Indeed, the prevalence of hypertension in survivors of childhood cancer exceeds 70% at the age of 50.^15^ This adds to the risk of developing CVD and long-term end-organ damage and increases mortality.^16,17^ Importantly, these detrimental vascular effects become increasingly relevant as many novel targeted therapies lead to durable anticancer responses, contributing to prolonged survival in patients with cancer.^16,17^ Thus, the prevention, identification, and prompt treatment of hypertension caused by antineoplastic agents is important to avert both short- and long-term adverse cardiovascular consequences.

This review highlights the interplay between cancer and hypertension and discusses the increased burden of CVD in patients with cancer. The incidence and pathogenesis of hypertension associated with a selection of predominantly targeted anticancer therapies, particularly inhibitors of the VEGF (vascular endothelial growth factor) pathway are reviewed. Finally, clinical strategies to screen, monitor, and treat hypertension in the oncology population are discussed.

## Interplay Between Cancer and Hypertension

CVD and cancer are the most common causes of morbidity and mortality in the developed world.^18,19^ Both classes of disease share numerous, potentially modifiable risk factors, including increased body mass index, diabetes,^20^ and tobacco use.^21,22^ Notably, most of these shared risk factors are also associated with the development of hypertension. Population studies suggest that hypertension is at least partly attributable to obesity in around 78% of cases,^23,24^ and up to 80% of patients with type 2 diabetes develop hypertension.^25^ Importantly, a large observational cohort study demonstrated that hypertension is the most common comorbidity in patients with cancer, with a reported prevalence of 38%.^26^ As this study was published before the widespread introduction of many targeted therapies associated with hypertension, this is likely to be an underestimate of the current prevalence of hypertension among patients with cancer.

### Parallel Pathophysiological Mechanisms in Cancer and Hypertension

**Figure 1.**
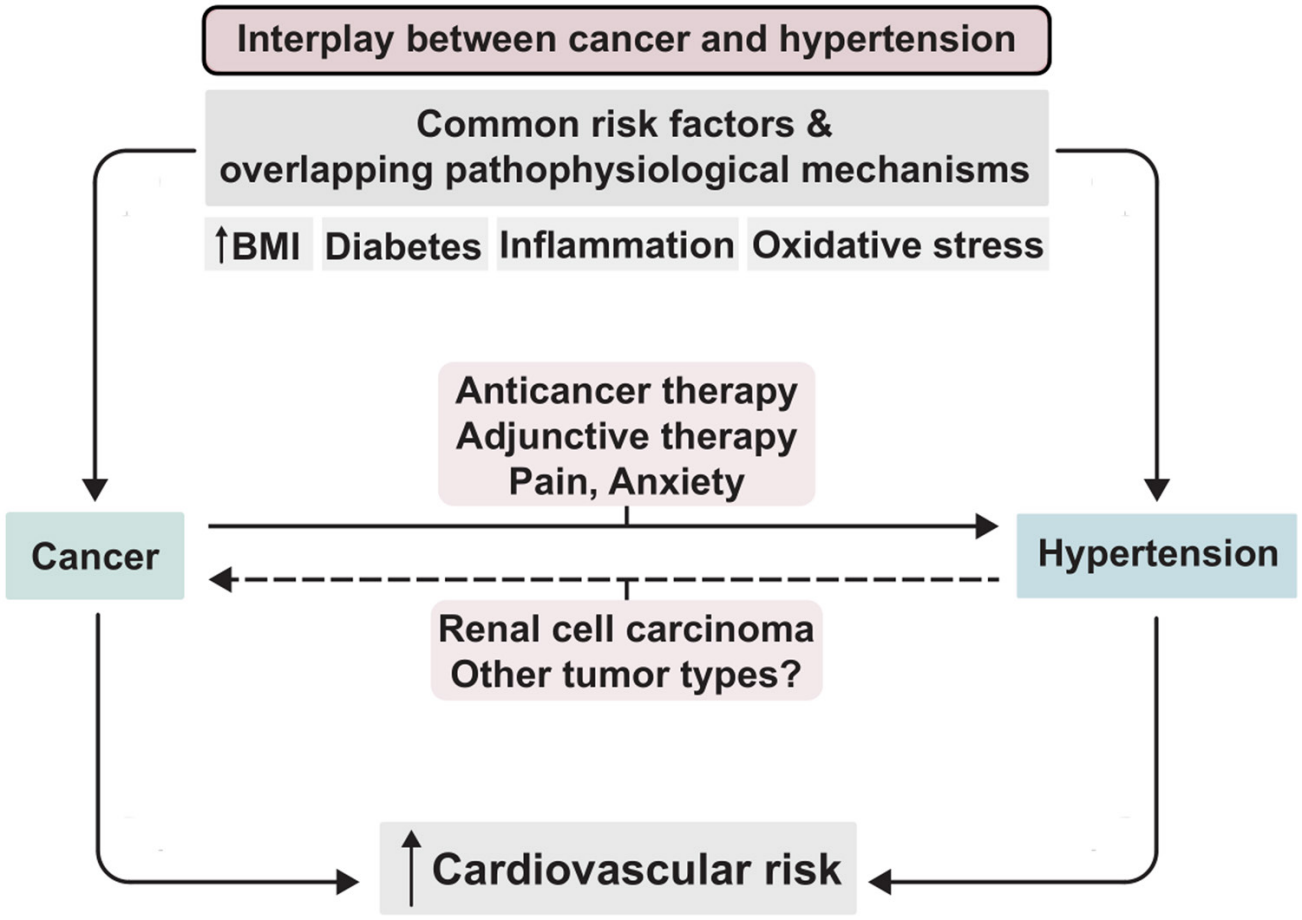
**The interplay between cancer and hypertension.** Cancer and hypertension frequently occur in the same patients, which is partly attributable to common risk factors and overlapping pathophysiological mechanisms for both conditions, including an increased body mass index (BMI), diabetes, inflammation, and oxidative stress. Furthermore, various anticancer therapies and adjunctive therapies exert prohypertensive effects, predisposing cancer patients to the development of hypertension. This risk could be further increased due to cancer-associated factors, such as inadequate pain control and anxiety. A direct relationship between hypertension and renal cell carcinoma has been proposed, but this has not been verified for other malignancies. Eventually, this interplay contributes to the elevated cardiovascular risk observed in patients with cancer.

The fact that cancer and hypertension frequently co-occur and share multiple risk factors suggests that overlapping pathophysiological mechanisms play prominent roles in both conditions. The search for overlapping mechanisms involved in the pathogenesis of both conditions has highlighted important processes (Figure 1), including inflammation and an increase in reactive oxygen species (ROS) and oxidative stress.

Inflammatory cells and cytokines are important constituents of the tumor microenvironment and targeting inflammatory mediators such as tumor necrosis factor-α and interleukin-1β reduces the incidence and spread of cancer.^27^ Similarly, inflammatory cell infiltration is observed within the renal interstitium and the arterial vascular wall of hypertensive rats^28,29^ and inhibition of these inflammatory processes ameliorates hypertension.^30^ Clinical data from the Global Cancer Incidence, Mortality, and Prevalence 2018 database, which gathers data from 185 countries, estimated that 13% of all cancer diagnoses were attributable to chronic infections.^31^ Similar to the hypothesis that inflammatory activation may predispose to the development of cancer, elevated baseline serum levels of inflammatory markers, including C-reactive protein and interleukin-6, were associated with a subsequent diagnosis of hypertension in a study of 20 525 American women.^32^ A comparable association between baseline inflammatory status and the subsequent development of hypertension has been observed in a meta-analysis of 142 640 patients recruited to cohort or nested case-control studies.^33^

In mice, downregulation of the tumor suppressor p53 (mutated in ≈ 50% of malignancies) is associated with increased levels of oxidative stress and production of ROS. P53 knockout mice displayed a high subsequent incidence of spontaneous lymphoma and accelerated growth of xenograft tumors.^34^ Notably, the antioxidant N-acetylcysteine was an effective inhibitor of tumor growth. These data suggest that ROS play an important role in tumor development, and that ROS production may, at least partly, be regulated by p53.^34^ Furthermore, extensive experimental data from a variety of hypertensive models demonstrate the role of ROS and oxidative stress in the development of hypertension.^35^ However, the benefits of targeting oxidative stress in patients are not well-established. A study in male physicians found that long-term supplementation of antioxidant multivitamins was modestly effective in reducing the incidence of total cancer (a composite outcome consisting of multiple cancer subtypes). However, this protective effect was only present in individuals with a baseline history of cancer and not in the much larger group without previous cancer.^36^ In contrast, a recent study in patients with breast cancer demonstrated that antioxidant supplements may be associated with an increased chance of breast cancer recurrence, possibly by reducing the cytotoxicity of chemotherapy.^37^ Also, the preventive effects of antioxidant supplementation on the prevention of mortality from various diseases, including CVD and cancer, was not verified by a large Cochrane meta-analysis.^38^ Thus, despite these proposed roles of ROS in the development of cancer and hypertension, ROS modulation is currently not an established clinical treatment for the prevention or treatment of either condition.

### Hypertension As a Possible Risk Factor for Cancer

Although hypertension and cancer have overlapping risk factors, studies investigating the direct associations between hypertension and incident cancer have been largely inconsistent.^39,40^ Hypertension has been proposed as an independent risk factor for renal cell carcinoma (RCC) in several observational studies.^39–42^ One study of almost 300 000 patients examined the relationship between blood pressure, antihypertensive medication, and RCC within the European Prospective Investigation into Cancer and Nutrition study population.^43^ Over a mean follow-up of 6.2 years, patients with systolic blood pressure (SBP) ≥160 mmHg or diastolic blood pressure ≥100 mmHg had a 2.5-fold increased risk of developing RCC compared with patients with SBP <120 mmHg or diastolic blood pressure <80 mmHg.^43^ Notably, an association between antihypertensive therapy and cancer was only found when blood pressure was poorly controlled, suggesting that high blood pressure itself may predispose these individuals to the development of RCC. An alternative explanation could be that a confounding factor predisposes these patients to both cancer and hypertension that is difficult to control. However, the association between hypertension and the incidence of RCC was further verified in a large population cohort study among almost 10 million South Korean adults. Hypertensive individuals had an increased incidence of RCC (20.9 versus 9.2 cases per 100 000 person-years, respectively) after a follow-up of 8 years with an adjusted hazard ratio of 1.12.^41^ The underlying mechanisms predisposing hypertensive individuals to developing RCC are thought to involve hypertension-induced chronic kidney disease, inflammation, and upregulation of oncogenic hypoxia-inducible factors and ROS.^41,42^ Furthermore, in conjunction with hypertension, other risk factors such as obesity, may be important in the development of RCC.^42,44^

In contrast to RCC, the association between hypertension and the incidence of other malignancies is less clear. Several studies have suggested a link between hypertension and breast cancer, particularly in postmenopausal women.^39,45^ In a meta-analysis of 30 prospective studies, hypertension was associated with a 20% increased breast cancer risk in postmenopausal women,^45^ but this association was not confirmed in a large Taiwanese population study including 111 000 individuals.^46^ Also, links between hypertension and colorectal, endometrial, prostate, and hepatocellular cancer have been proposed, but studies demonstrating a clear causal relationship are lacking.^39,47,48^ Importantly, other studies suggest that hypertension has little or no association with several other cancer types, including malignancies of the stomach, gallbladder, pancreas, and lung.^39,40,49,50^

### Increased Cardiovascular Risk in Cancer Survivors

Improved cancer survival has exposed a range of late adverse cardiovascular effects.^51^ This has been particularly evident following childhood malignancy, as over 80% of children now survive at least 5 years after cancer diagnosis.^51^ Notably, these childhood and adolescent cancer survivors have a 7-fold increased risk of cardiovascular death when compared with the general population,^52^ and this elevated risk persists beyond 50 years of age.^53^ Also, with the exception of recurrent cancer, CVD is the leading cause of morbidity and mortality in this population.^54^ This increased tendency to develop CVD is also observed in survivors of adult-onset cancers, as demonstrated in a large registry analysis of 3.2 million cancer survivors in the United States.^55^ The risk of developing CVD increases over time and CVD overtakes breast cancer as the primary cause of mortality in older breast cancer survivors 10 years after the initial diagnosis.^56^

The marked increase in the risk for CVD may partly be explained by the higher prevalence of hypertension in patients undergoing cancer treatment or who have survived in the medium to long term. Indeed, in a study of ≈3000 adult 10-year survivors of childhood cancer, the prevalence of hypertension exceeded 70% by 50 years of age and was 2.6-fold higher than would be expected based upon age-, sex-, race-, and body mass index-specific rates in the general population.^15^ Additionally, a retrospective study found that these survivors are more likely to be prescribed antihypertensive medication than their siblings (odds ratio 1.6).^57^ This increased risk for the development of hypertension both in the short and longer term is likely to be primarily related to anticancer therapies. In a retrospective analysis of over 25 000 adult cancer patients in the United States, approximately one-third of patients developed new-onset hypertension during follow-up, and anticancer therapy was associated with a 2- to 3.5-fold increased risk of hypertension.^58^ Furthermore, the large Childhood Cancer Survivor Study demonstrated that the presence of hypertension in cancer survivors increased the relative risk (RR) of cardiac events, including coronary artery disease (RR, 6.1), heart failure (RR, 19.4), valvular disease (RR, 13.6), and cardiac arrhythmias (RR, 6.0) independent of cancer therapy–related risk.^16^ Of note, the risks to develop these major cardiac events were even higher in survivors who developed other cardiovascular risk factors or who had received anthracyclines or chest radiotherapy.^16^ These findings were supported by a study of 23 000 5-year survivors from the same cohort.^59^ Fortunately, advances in cancer therapies and better health surveillance programs have reduced toxicities related to anticancer therapy in children, which has also improved their cardiovascular outcomes.^59^ Nonetheless, hypertension remains an important determinant of the increased risk of cardiac events in patients with cancer and cancer survivors, in whom hypertension can be preexisting or develop de novo in the context of cancer therapy.^16,60^

## Anticancer Therapy and Hypertension

**Figure 2.**
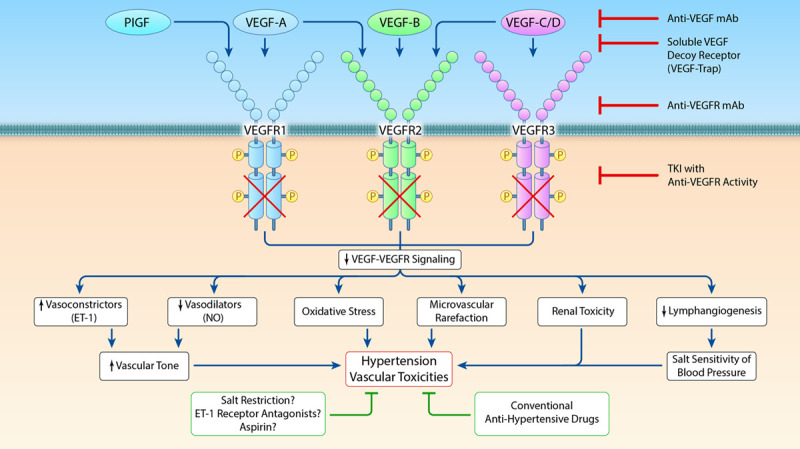
**Pathophysiological mechanisms underlying VEGF (vascular endothelial growth factor) inhibitor (VEGFI)-induced hypertension and possible therapeutic interventions.** Clinically, four different major classes of agents to inhibit VEGF signaling can be distinguished: (1) monoclonal antibodies directed against circulating VEGF; (2) soluble decoy receptors (VEGF-traps), scavenging freely available VEGF; (3) monoclonal antibodies against the vascular endothelial growth factor receptor (VEGFR); (4) TKI with anti-VEGFR activity that act on the intracellular tyrosine kinase domains of VEGFR to inhibit their activation. Multiple mechanisms contribute to VEGFI-induced hypertension, including an imbalance between vasoconstrictor (ET-1 [endothelin-1]) and vasodilator factors (nitric oxide [NO]), oxidative stress, microvascular rarefaction, renal injury, and decreased lymphangiogenesis. Conventional antihypertensive drugs, including calcium channel blockers and angiotensin-converting enzyme inhibitors / angiotensin II receptor blockers can be used in the treatment of VEGFI-induced hypertension. Additional potential treatment options include salt restriction, ET-1 receptor antagonists and aspirin. However, ET-1 receptor antagonists are currently not registered for the treatment of systemic hypertension. mAb indicates monoclonal antibody; PlGF, placental growth factor; and TKI, tyrosine kinase inhibitor (Illustration credit: Ben Smith).

**Figure 3.**
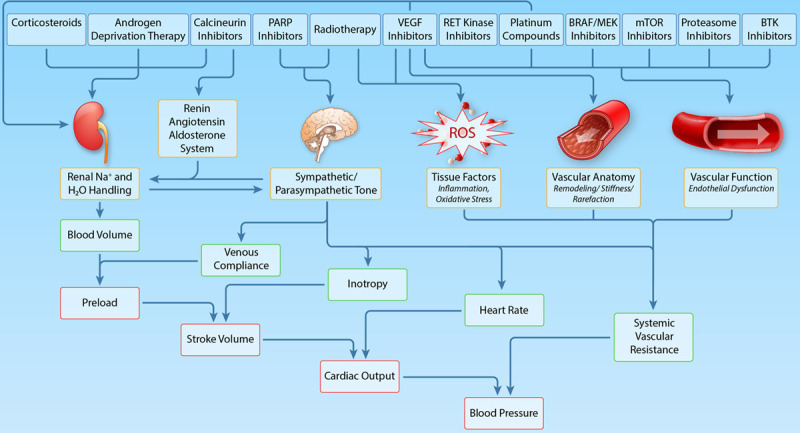
**Hypothesized mechanisms underlying the prohypertensive effects of various classes of antineoplastic drugs and adjuvant therapies (Illustration credit: Ben Smith).** BRAF indicates v-raf murine sarcoma viral oncogene homolog B1; BTK, Bruton's tyrosine kinase; MEK, mitogen-activated protein kinase kinase; mTOR, mammalian target of rapamycin; PARP, poly ADP ribose polymerase; RET, rearranged during transfection; and VEGF, vascular endothelial growth factor.

In addition to possible pathophysiological associations between cancer and hypertension, a wide range of anticancer compounds and adjunctive therapies used in oncology have been shown to have prohypertensive effects. Awareness of hypertension induced by antineoplastic agents largely arose following the introduction of vascular endothelial growth factor inhibitors (VEGFI), which are associated with hypertension in a large proportion of treated individuals.^5^ Additionally, many other commonly used antineoplastic agents have been associated with an increase in blood pressure and either de novo hypertension or a deterioration of previously well-controlled hypertension. Notably, patients with comorbidities such as CVD and uncontrolled blood pressure are frequently excluded from oncological clinical trials. Therefore, these sources of data underestimate the true incidence of hypertension and other cardiovascular toxicities.^4,61–63^ For most antineoplastic agents, the evidence regarding their prohypertensive effects is primarily derived from observational and retrospective clinical studies. Also, the pathophysiological mechanisms by which these compounds lead to an increase in blood pressure are mainly based on observations from preclinical and in vitro studies, rather than from clinical studies or trials. Nonetheless, next to VEGFI (Figure 2), the prohypertensive effects of a selection of predominantly novel orally administered targeted therapies are subsequently discussed, based on the available evidence from previous literature.^7,64–67^ The mechanisms underlying these prohypertensive effects are displayed in Table 1 and Figure 3.

**Table 1.**
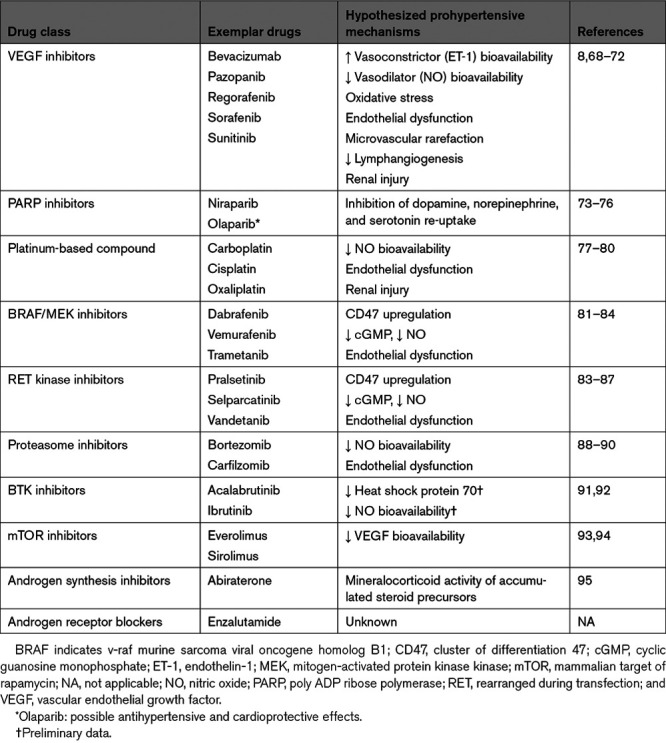
Anticancer Agents With Marked Prohypertensive Effects

### Vascular Endothelial Growth Factor Inhibitors

VEGFI are used as anticancer treatment in a wide range of malignancies, particularly in the metastatic setting. VEGFI exploit the dependency of tumors upon blood supply by inhibiting angiogenesis, the formation of new blood vessels from preexisting vasculature. Angiogenesis is predominantly mediated by VEGF, which is secreted by various cell types, including endothelial cells, fibroblasts, and tumor cells. In humans, the VEGF family consists of 5 structurally related dimeric proteins: VEGF-A, VEGF-B, VEGF-C, VEGF-D, and PlGF (placental growth factor).^96^ VEGF acts by binding to one of its 3 tyrosine kinase receptors (vascular endothelial growth factor receptor [VEGFR]1, 2, and 3), of which VEGFR2 is the main signaling VEGFR (Figure 2).^97^ Activation of the VEGFR leads to multiple downstream effects, including an increase in capillary permeability, production of the vasodilator nitric oxide (NO) and promotion of vascular endothelial cell survival.^98^ In addition to promoting angiogenesis, VEGF also plays an important role in several other physiological processes, including lymphangiogenesis, the menstrual cycle, and wound healing.^99^ As depicted in Figure 2, four main types of VEGFI can be distinguished based on their mechanism of action.^8^ Notably, many tyrosine kinase inhibitors (TKI) with anti-VEGF activity interfere not only with VEGF signaling but also with alternative (proangiogenic) growth factors and receptors, such as the platelet-derived growth factor, fibroblast growth factor, c-Kit and Flt-3.^100^

Although VEGFI have led to a marked improvement in outcomes in various malignancies such as metastatic RCC,^101^ advanced hepatocellular carcinoma,^102^ cervical cancer,^103^ and gastrointestinal stroma cell tumors,^104^ these antineoplastic agents are associated with a range of unwanted cardiovascular effects.^4,6,8^ These include hypertension, left ventricular systolic dysfunction, arterial and venous thrombosis, and cardiac arrhythmias.^105–107^ Hypertension is the most frequently encountered side-effect and occurs in 20% to 90% of treated patients, depending on VEGFI type and dosage.^8,100^ However, other studies reported lower incidences of hypertension (≈20%–40%).^5,108^ A large meta-analysis of 29 000 patients with cancer demonstrated that patients receiving VEGF-TKI treatment had a RR of 3.78 for the development of hypertension compared with the control group.^105^ A complicating factor in determining the precise incidence and severity of VEGFI-induced hypertension is that these studies used different versions of the National Cancer Institute’s Common Terminology Criteria for Adverse Events to define hypertensive events.^8^ Low-grade VEGFI-induced hypertension (grade 1 or 2) occurs the most frequently and almost every patient experiences a rapid increase in baseline blood pressure within a few days after initiation of VEGFI therapy.^61,68^ Nonetheless, a substantial proportion of treated patients develop high-grade hypertension (grade 3 or 4), ranging from 6% to 43%.^8^

The extent of the VEGFI-induced rise in blood pressure is dose-dependent and blood pressure normalizes quickly upon drug withdrawal.^69,109^ Therefore, it is proposed that this hypertensive response reflects treatment efficacy and represents an on-target mechanism.^110^ Indeed, retrospective studies in patients with metastatic RCC or gastrointestinal stroma cell tumors demonstrated that the development of hypertension during administration of the VEGF-TKI sunitinib was predictive of improved survival outcomes compared with patients who remained normotensive.^62,111^ This relationship between VEGFI-induced hypertension and improved survival has not been demonstrated for all tumor types.^112^ Importantly, 2 studies demonstrating the association between hypertension and improved cancer survival outcomes found that the use of antihypertensive agents or effective antihypertensive prophylaxis did not impair antitumor treatment effectivity.^62,113^

### Mechanisms Leading to VEGFI-Induced Hypertension

While the exact mechanisms underlying the hypertensive effects of VEGFI remain elusive, several molecular mechanisms have been proposed (Figure 2). VEGF is an important modulator of vascular tone and blood pressure,^114^ as demonstrated by a clinical study in which administration of recombinant human VEGF in patients with coronary artery disease led to a rapid decrease in mean arterial pressure.^115^ Normally, VEGF increases the production of NO and prostacyclin, 2 vasodilators, by vascular endothelial cells via stimulation of eNOS (endothelial NO synthase) activity and cytosolic phospholipase A2-mediated arachidonic acid release, respectively.^116,117^ Consequently, VEGFI decrease the bioavailability of these vasodilators.^118,119^ On the other hand, VEGFI have been demonstrated to markedly increase the bioavailability of vasoconstrictors, particularly ET-1 (endothelin-1).^69^ Therefore, this causes an imbalance between vasodilators and vasoconstrictors skewed toward the latter. This enhances vasomotor tone and contributes to the development of hypertension. Polymorphisms in eNOS that are suggested to decrease eNOS activity and consequently lead to decreased plasma NO levels were associated with the development of high-grade hypertension during sunitinib therapy.^120^ This further stresses the importance of a delicate balance between vasoconstrictor and vasodilator factors. Interestingly, a preclinical study demonstrated that ET-1 receptor antagonists attenuate the sunitinib-induced rise in blood pressure. However, these agents are currently not approved for the treatment of systemic hypertension in humans.^121^

Oxidative stress has been implicated as an additional important contributor to the hypertensive effects of VEGFI by inducing endothelial dysfunction, as demonstrated by elevated levels of ROS in VEGFI-treated rats.^70,122^ Interestingly, recent evidence indicates that the increase in ET-1-mediated vasopressor response and ROS generation could be mediated by circulating endothelial nanoparticles as a result of VEGFI-associated endothelial injury.^123^ However, administration of Tempol, a ROS scavenger, did not lead to a relevant attenuation of sunitinib-induced increase in blood pressure in a preclinical model.^121^

Microvascular rarefaction (a reduction in microvessel density), leading to impaired microcirculation and increased vascular resistance, has been proposed to contribute to VEGFI-induced hypertension, given that a moderate degree of rarefaction has been observed in patients receiving VEGFI.^71^ However, this rarefaction is most likely functional, rather than structural, given the rapidity of blood pressure increase after initiation of therapy and the quick blood pressure normalization after VEGFI withdrawal. Of note, an increase in vascular stiffness has been reported during VEGFI therapy. A study in 84 patients with metastatic RCC demonstrated that sunitinib increased large artery stiffness within the first weeks of therapy, as measured by increased carotid-femoral pulse wave velocity.^124^ This increase in vascular stiffness was also observed after 3 weeks of sorafenib treatment in another clinical study.^125^ Nonetheless, the exact contribution of vascular stiffness to the prohypertensive effects of VEGFI remains unclear, as it could both be a cause and consequence of hypertension.^125,126^

It is notable that there is scarce evidence for a role of the renin-angiotensin-aldosterone system (RAAS) in VEGFI-associated hypertension. Indeed, previous clinical studies demonstrated that plasma renin levels decreased during VEGFI therapy, indicative of suppressed RAAS activity.^69,125^ Furthermore, aldosterone levels remained unchanged during VEGFI treatment, while the development of hypertension in a patient who previously underwent a bilateral adrenalectomy suggested that aldosterone is not indispensable for developing hypertension in this context.^125,127^ In a preclinical study, the angiotensin-converting enzyme inhibitor (ACEI) enalapril did not prevent VEGFI-induced hypertension but ameliorated VEGFI-induced renal toxicity.^128^ One retrospective analysis of patients with metastatic RCC enrolled in clinical trials of anticancer therapy reported that survival outcomes were better for patients with hypertension treated with RAAS inhibitors in comparison to those treated with other antihypertensive agents.^129^ Although these findings are notable, they may be confounded by treatment selection bias.

VEGFI-induced hypertension is salt-sensitive^68,130^ as demonstrated by a preclinical study in which VEGFI-induced rise in blood pressure was further increased by administration of a high-salt diet, accompanied by a rightward shift of the renal pressure-natriuresis curve.^72^ Impaired salt buffering in the skin due to inhibition of VEGF-C-mediated lymphangiogenesis during angiogenesis inhibition has been proposed to underlie this salt-sensitive hypertension, but this concept is not supported by investigational data.^72^ A current clinical trial is investigating the effects of salt restriction on the blood pressure levels in VEGFI-treated patients (Netherlands Trial Register: URL: https://www.trialregister.nl; Unique identifier: NL7340).

### VEGFI-Induced Renal Toxicity

VEGF plays an important role for the maintenance of a healthy fenestrated endothelium in the renal glomerular apparatus, and VEGFI treatment can have nephrotoxic effects.^131^ Izzedine et al propose that 2 main types of VEGFI-induced renal events can be distinguished, corresponding to the type of VEGFI therapy used. In a series of renal biopsies in patients with VEGFI-associated renal toxicity, anti-VEGF-ligands (anti-VEGF monoclonal antibodies and soluble VEGF decoy receptors) were predominantly associated with thrombotic microangiopathy, whereas VEGF-TKI were mainly associated with minimal change nephropathy and/or focal segmental glomerulosclerosis.^132,133^ These renal toxicities may lead to proteinuria and sodium and water retention, further contributing to the rise in blood pressure observed during VEGFI therapy.^8^

The adverse vascular and renal effects occurring during VEGFI have been termed a preeclampsia-like syndrome as these resemble the hallmarks of the severe pregnancy complication preeclampsia.^121^ Preeclampsia is characterized by hypertension, proteinuria, and increased plasma levels of sFlt-1 (soluble fms-like tyrosine kinase-1), a soluble VEGFR. As a consequence, VEGF bioavailability is greatly diminished in preeclamptic women, and this is thought to play an important role in the pathogenesis of this disease.^134^ Of note, the cyclo-oxygenase inhibitor aspirin is an effective preventive strategy for preeclampsia in high-risk women as it is thought to restore the imbalance between vasoconstrictor and vasodilator factors.^135^ In this context, aspirin also has the potential to ameliorate the adverse effects of VEGFI therapy. Indeed, the beneficial effects of aspirin were verified by a recent study in sunitinib-treated rats, but clinical benefits in VEGFI-treated patients awaits verification.^122^

### VEGFI-Induced Cardiac Toxicity

Treatment with VEGFI is also associated with left ventricular systolic dysfunction and heart failure.^136,137^ The clinical spectrum of VEGFI-associated cardiotoxicity ranges from asymptomatic left ventricular dysfunction and mild QT_c_-interval prolongation, to heart failure, cardiogenic shock, and death.^138,139^ One study prospectively monitored 90 RCC patients receiving sunitinib via echocardiography and cardiac biomarkers.^140^ When compared with baseline, 10% of these patients had a reduction in left ventricular ejection fraction of ≥10% to a value <50%, with the majority of these changes occurring within the first treatment cycle. Importantly, left ventricular dysfunction was at least partially reversible with sunitinib dose modification and/or treatment with antihypertensive medication.^140^ VEGFI have the potential to induce direct myocardial toxic effects and consequently reduce the heart’s capacity to withstand a rise in afterload as a result of concurrent systemic hypertension.^141^ This further highlights the need for adequate cardiovascular monitoring and blood pressure control both before and during VEGFI therapy. While beyond the scope of this review, a detailed overview of the mechanisms underlying VEGFI-associated cardiotoxicity has been published recently.^142^

### Poly ADP Ribose Polymerase Inhibitors

PARP (poly ADP ribose polymerase) inhibitors such as olaparib, niraparib, rucaparib, and talazoparib have been approved by the United States Food and Drug Administration for use in breast and ovarian malignancies.^73^ However, their efficacy has also been studied in pancreatic and biliary tract cancers, as well as glioblastoma, lung, and prostatic cancers.^143^ PARP inhibitors trap PARP1 and PARP2 at DNA damage sites and prevent the recruitment of additional DNA repair proteins. Consequently, during the replication of tumor cells, DNA repair is inhibited and apoptosis and cell death ensues.^144^

In this drug class, only niraparib has been associated with hypertension.^73^ In the NOVA trial (Niraparib Maintenance Therapy in Platinum-Sensitive, Recurrent Ovarian Cancer), any-grade and grade 3 or 4 hypertension occurred in 19% and 8% of patients treated with niraparib, respectively,^145^ versus 5% and 2%, respectively, in placebo-treated patients.^145^ The prohypertensive effects of niraparib could reflect an off-target effect: the Food and Drug Administration approval summary for niraparib states that it can bind to dopamine, norepinephrine, and serotonin transporters, inhibiting their cellular uptake, which is accompanied by a greater ability of niraparib to penetrate the central nervous system than other PARP inhibitors.^74^ This has been proposed to contribute to the prohypertensive effects but is only speculative and mechanisms underlying niraparib-induced hypertension remain poorly understood.^73^

A number of trials examined the anticancer effects of combining PARP inhibitors with other anticancer agents.^146,147^ The addition of VEGFI to PARP inhibition in patients with ovarian cancer has shown promising oncological effects, including longer progression-free survival when compared with PARP inhibition alone.^148,149^ This may, however, also increase the risk of hypertension, particularly in the case of niraparib. Indeed, in the phase 2 AVANOVA2 trial (Niraparib Plus Bevacizumab Versus Niraparib Alone for Platinum-Sensitive, Recurrent Ovarian Cancer), 56% of patients receiving a combination of niraparib and the VEGFI bevacizumab developed hypertension, compared with 22% of patients receiving niraparib monotherapy.^149^

As noted, other PARP inhibitors have not been associated with prohypertensive effects. In 46 patients with ovarian cancer, olaparib monotherapy was not associated with the development of hypertension.^148^ In fact, in the absence of confounding central effects, there is reasonable mechanistic evidence to suggest that these agents may also have the potential to confer protective effects on the heart and vasculature. Indeed, PARP inhibitors have been demonstrated to prevent cardiomyocyte necrosis and reduce myocardial infarction size after cardiac reperfusion injury and to protect against vascular endothelial dysfunction in animal models, including hypertensive and diabetic mice.^75,76^ Interestingly, the PAOLA-1 trial (Olaparib Plus Bevacizumab Versus Bevacizumab Alone Maintenance in Advanced Ovarian Cancer) of 806 patients reported a numerically lower incidence of hypertension in the olaparib and bevacizumab combination group compared with bevacizumab alone (46% versus 60%).^146^ Although the suggestion that PARP inhibition might confer clinically meaningful cardiovascular protective effects in patients with cancer is an intriguing hypothesis, this has not been adequately investigated.

### Platinum-Based Compounds

Platinum-based compounds (cisplatin, carboplatin, oxaliplatin) are widely used to treat testicular, ovarian, colorectal, bladder, and lung cancers as well as mesothelioma.^150^ Their anticancer mechanism of action involves DNA uptake of platinum, with subsequent induction of apoptotic cell death via inhibition of transcription.^151^ Hypertension is common following platinum-based chemotherapy, although the reported prevalence varies between trials.^152–154^ In contrast to hypertension seen with VEGFI, platinum therapy-associated hypertension tends to be a long-term effect, potentially occurring years after treatment. This is especially relevant in testicular cancer, which has a high survival rate and is the most common cancer in young men. One study of 1289 testicular cancer survivors reported that 53% of patients receiving a cumulative dose of over 850 mg cisplatin developed hypertension over a median follow-up of 11 years, with an odds ratio of 2.3 compared with healthy controls.^152^ Other studies, with follow-up ranging from 7 to 14 years, have reported a prevalence of hypertension ranging between 14% and 39%.^153,154^

These data suggest that hypertension develops and persists in a substantial proportion of patients following platinum-based therapy. Of note, cisplatin is detectable in serum up to 13 years after initial exposure, which may provoke chronic endothelial activation. Indeed, higher levels of circulating platinum have been associated with an increased risk of hypertension.^77^ In patients with a history of testicular cancer treated with cisplatin at least 10 years previously, microalbuminuria (closely linked to endothelial dysfunction) was found in 22% of patients.^154^

Endothelial cell activation and endothelial damage is thought to play an important role in the development of platinum-associated hypertension.^66,78^ Exposure of human umbilical vein endothelial cells to cisplatin resulted in upregulation of intercellular adhesion molecule-1, leading to increased leucocyte/endothelial interaction,^79^ while treatment of human dermal microvascular endothelial cells with cisplatin and bleomycin decreased endothelial cell survival and increased apoptosis.^78^ Furthermore, NO production was found to be reduced in human umbilical vein endothelial cells exposed to carboplatin and paclitaxel.^80^ Although these are in vitro observations which require verification in a clinical study, these mechanisms could all lead to endothelial dysfunction, contributing to hypertension. In addition to hypertension, platinum-based therapies also predispose to chronic kidney disease due to nephrotoxic effects and the development of metabolic syndrome in the long-term, further increasing CVD risk.^155^

### BRAF/MEK Inhibitors

Inhibitors of BRAF (v-raf murine sarcoma viral oncogene homolog B1) and inhibitors of MEK (mitogen-activated protein kinase kinase) are approved for use in patients with a range of malignancies, including melanoma, colorectal cancer, hepatocellular carcinoma, RCC, and gastrointestinal stroma cell tumors. These agents target BRAF (one of the three RAF kinase family members) and MEK, which are important kinases in the RAS-RAF-MEK-ERK (extracellular signal-regulated kinase) signaling pathway, which plays important roles in promoting cell proliferation, differentiation, and resistance to apoptosis.^156^ Activating BRAF mutations have been observed in up to 60% of melanomas, as well as in smaller proportions of other malignancies.^157^ Multitargeted TKI, such as sorafenib and regorafenib, target BRAF and its mutant forms as well as VEGFR tyrosine kinases. These agents have been associated with a high prevalence of hypertension, which may be a result of VEGF inhibition as well as their effects on BRAF. In clinical trials, the incidence of hypertension in patients treated with more specific BRAF inhibitors, such as vemurafenib and dabrafenib, varied from 6% to 14%.^158,159^

Unfortunately, treatment resistance to BRAF inhibition occurs frequently, which may be due to increased downstream MEK activation.^160^ Therefore, MEK inhibitors, including trametinib, were developed. Combination therapy of BRAF and MEK inhibitors has improved outcomes for patients with melanoma.^161^ MEK inhibitors have also been associated with hypertension. In the METRIC trial (MEK Inhibition Versus Chemotherapy in Advanced or Metastatic BRAF-Mutant Melanoma) of 322 patients, the incidence of grade 2 to 3 hypertension was 15% in the trametinib group versus 7% in the chemotherapy group.^162^ Additionally, a meta-analysis of 2704 patients treated with MEK inhibitors, either as monotherapy or in combination with a BRAF inhibitor, reported a RR of 1.5 for the development of hypertension compared with controls treated with alternative agents.^163^ Additionally, in the COMBI-d trial (Dabrafenib and Trametinib Versus Dabrafenib and Placebo in Patients With BRAF-Mutant Melanoma) of 423 patients, any-grade hypertension was more common in the combination treatment group compared with the dabrafenib monotherapy group (22% versus 14%).^158^ However, high-grade hypertension (SBP ≥160 mm Hg or diastolic blood pressure ≥100 mm Hg) was similar between both groups (4% versus 5%).^158^ Similarly, a meta-analysis including 2317 patients treated with combination BRAF/MEK inhibitor therapy reported an incidence of any-grade hypertension of 20% compared with 14% in controls treated with BRAF inhibitor monotherapy (RR 1.5).^81^ This study also reported an overall incidence of high-grade hypertension of 8% in combination therapy compared with 5% in the control group.^81^

Although the mechanisms leading to BRAF/MEK inhibitor-induced hypertension are incompletely defined, studies in cancer cell lines could provide some insight. The upregulation of CD47 (cluster of differentiation 47) appears to be of central importance. CD47 expression is frequently increased in tumors^164^ and interacts with phagocytic cells to prevent cancer cell phagocytosis.^165^ In cultured melanoma cells treated with BRAF/MEK inhibitors, rebound ERK activation induces upregulation of CD47 via the transcription factor nuclear respiratory factor-1.^82^ CD47 subsequently inhibits NO bioavailability and NO-induced activation of sGC (soluble guanylate cyclase), thereby reducing levels of the vasodilator cGMP (cyclic guanosine monophosphate).^83,84^ This sequence of events might translate to endothelial dysfunction, increased vascular constriction, and subsequent hypertension in vivo. Nonetheless, these consequences of CD47 upregulation have been demonstrated in melanoma cells only and further studies on endothelial cells and vascular smooth muscle cells are required.^84^

### RET Kinase Inhibitors

Mutations in RET (rearranged during transfection), a receptor tyrosine kinase, are found in thyroid cancer and nonsmall cell lung cancer and present a potential therapeutic target.^166^ Several Food and Drug Administration-approved multikinase inhibitors including vandetanib and cabozantinib have activity against RET; however, none have been approved solely on the basis of their anti-RET kinase action. More recently, the selective RET kinase inhibitors selpercatinib and pralsetinib have been approved for use in patients with RET mutations in these malignancies.^85,86,167^ In a phase 1 to 2 study of 162 patients with RET-mutant medullary thyroid or RET fusion-positive thyroid cancer treated with selpercatinib, 43% developed any grade hypertension. Of note, 21% of patients developed blood pressure ≥160/100 mm Hg.^86^ Similarly, in 105 patients with nonsmall cell lung cancer, 31% of patients treated with selpercatinib developed any grade hypertension.^85^

To the best of our knowledge, mechanisms underlying RET inhibitor-associated hypertension have not been studied. However, given the role RET kinase plays within the RAS-RAF-MEK-ERK pathway,^87^ RET inhibition may lead to rebound ERK activation similar to that seen with BRAF/MEK inhibition. Thus, CD47 upregulation may also be important in the development of hypertension with RET inhibitors. Recent studies of biopsies taken from patients treated with RET inhibitors have identified amplification of K-RAS, a member of the RAS family of proteins, as a potential source of resistance to these agents, which could be indicative of rebound ERK activation.^168^

### Proteasome Inhibitors

Proteasome inhibitors (bortezomib, carfilzomib), used for the treatment of multiple myeloma and mantle cell lymphoma, induce tumor cell death by disrupting the activity of the proteasome. Proteasome inhibitors have been reported to have prohypertensive effects: a clinical trial among 929 multiple myeloma patients demonstrated that carfilzomib led to a longer median progression-free survival than bortezomib. However, this was at the cost of a higher incidence of all-grade (25% versus 14%) and high-grade (9% versus 3%) hypertension. This suggests a correlation between the prohypertensive effects and the efficacy of these agents.^169,170^ Studies addressing the underlying pathophysiology point to decreased NO bioavailability, causing endothelial dysfunction.^88^ Also, chronic proteasome inhibition was associated with impaired endothelium-dependent vasorelaxation in a preclinical study, indicative of endothelial dysfunction.^89^ These insights are mainly derived from in vitro and animal studies. Not surprisingly, the vascular effects are dose- and treatment duration-dependent.^90^

### BTK Inhibitors

Inhibitors of the BTK (Bruton’s tyrosine kinase) are used in various B-cell malignancies, including chronic lymphocytic leukemia and mantle cell lymphoma. In a study including 500 patients receiving the BTK inhibitor ibrutinib, 71% of patients who were normotensive at baseline developed new hypertension and 83% of patients with baseline hypertension experienced worsening of hypertension.^171^ However, another study in patients with chronic lymphocytic leukemia reported an incidence of ibrutinib-induced hypertension of 20% over a median follow-up of 29 months.^172^ Nonetheless, BTK inhibitors are frequently administered for prolonged periods of time, and this increased tendency to develop hypertension contributes to long-term cardiovascular risk. Careful monitoring of blood pressure during BTK inhibitor therapy is essential, as new or worsened BTK inhibitor-induced hypertension was associated with an elevated risk of developing major adverse cardiovascular events (hazard ratio 2.17), such as cardiac arrhythmias and myocardial infarction.^171^ Preliminary evidence suggests a possible correlation of BTK inhibitor-induced hypertension and a decrease in heat shock protein 70 downstream of the toll-like receptor-BTK signaling pathway.^91^ Also, inhibition of phosphatidylinositol 3-kinase-dependent NO production has been proposed, but the exact mechanisms underlying BTK inhibitor-induced hypertension have not been characterized.^92^

### mTOR Inhibitors

Inhibitors of mTOR (mammalian target of rapamycin), including everolimus and sirolimus, are mainly used to reduce the risk of organ rejection after organ transplantation. However, they remain in use in the treatment of several malignancies including RCC where they have become third-line treatment options.^173^ In everolimus-treated metastatic RCC patients, 10% developed any-grade hypertension.^93^ Normally, mTOR is a potent stimulator of VEGF secretion via hypoxia-inducible factor-1α.^94^ Consequently, inhibition of mTOR leads to decreased VEGF secretion, which may have prohypertensive effects similar to VEGFI.^94^ Indeed, other side effects of mTOR inhibition, such as podocyte damage and proteinuria, resemble VEGFI-induced toxicity.^174^ Of note, combining everolimus with the VEGF-TKI lenvatinib in patients with metastatic RCC was associated with all-grade and high-grade hypertension in 41% and 14% of patients, respectively.^93^ Furthermore, mTOR inhibitors impair glucose and lipid metabolism,^175^ and this may also contribute to the cardiovascular risk associated with these agents.

### Endocrine Therapy (Antiandrogens/Aromatase Inhibitors)

Androgen deprivation therapy prolongs overall survival in patients with metastatic prostate cancer by blocking the trophic effects of androgens on prostate cancer cells.^176^ This therapy concerns gonadotropin-releasing hormone agonists and therapies directly inhibiting androgen production and signaling. Gonadotropin-releasing hormone agonists may adversely affect traditional cardiovascular risk factors by elevating serum lipid levels, decreasing insulin sensitivity and promoting obesity, without displaying direct hypertensive effects.^177^ The androgen synthesis inhibitor abiraterone and the androgen receptor blocker enzalutamide have both been associated with hypertension. A large meta-analysis evaluated the cardiovascular toxicities of abiraterone and enzalutamide in 8660 prostate cancer patients. All-grade and high-grade hypertension was observed more frequently in the abiraterone group (26% and 7%, respectively) compared with placebo (15% and 4%, respectively), and the same was true for the enzalutamide group (11% and 5%, respectively) compared with placebo (4% and 2%, respectively).^178^ Importantly, these hypertensive events occurred irrespective of corticosteroid use, which is often administered concomitantly. Abiraterone inhibits testosterone production via inhibition of the cytochrome P450 (CYP17) enzyme with consequent accumulation of mineralocorticoid precursors. This effect contributes to the prohypertensive effects of abiraterone and can usually be successfully managed with a mineralocorticoid receptor antagonist.^95^ The mechanisms underlying enzalutamide-induced hypertension are still unclear.

### Adjunctive Therapy During Cancer Treatment

Adjunctive therapies, such as corticosteroids, EPO (erythropoietin), nonsteroidal anti-inflammatory drugs, calcineurin inhibitors, and radiotherapy are often administered concurrently with antineoplastic agents. These therapies can contribute to the development of hypertension or worsening of previously controlled hypertension.^65,179^ Therefore, careful monitoring is warranted when these are part of the anticancer therapy regime, particularly when co-administered with antineoplastic agents known to be associated with a rise in blood pressure.

Corticosteroids are an essential adjunctive therapy to many chemotherapy regimens for both hematologic malignancies and some solid tumors.^180,181^ They increase the efficacy of some antineoplastic agents via mechanisms that are mostly unclear. Also, they reduce treatment-associated side-effects and are frequently used as a form of palliative care.^180,181^ However, corticosteroids can lead to other significant side effects and a notable rise in blood pressure in conjunction with water and sodium reabsorption via mineralocorticoid receptor stimulation.^182^

EPO, in routine use to correct anemia caused by the underlying malignancy or anticancer therapy, has prohypertensive effects. Several mechanisms underlie EPO-induced hypertension, including increased blood viscosity, and, potentially, a skewed balance between vasoconstrictor and vasodilator prostaglandins with vascular resistance to the vasodilator effects of NO.^183^ Indeed, in rats treated with EPO, elevated vascular intracellular calcium concentrations were observed, which associated with vasoconstrictor effects that could not be compensated by cGMP upregulation.^184^

The modest prohypertensive effects of analgesic nonsteroidal anti-inflammatory drugs are well documented. These agents should be used with caution, especially in patients with hypertension, preexisting CVD, or in those receiving other treatments with potential cardiovascular toxic effects.^185,186^ Water and salt retention and decreased production of vasodilatory prostaglandins are thought to underlie the prohypertensive effects.^187^ Although there is currently insufficient evidence to suggest the routine use of low-dose aspirin for the prevention of VEGFI-associated hypertension, given the beneficial role of aspirin in the treatment of preeclampsia and the similarities between VEGFI-associated hypertension and preeclampsia, this is worthy of future investigation (Figure 2).^121,134,135^

Calcineurin inhibitors are used to prevent rejection of transplanted solid organs via inhibition of T cell function. In the field of hematology, calcineurin inhibitors are administered for the prevention of graft versus host disease in the context of allogenic bone marrow transplantation. Calcineurin inhibitors have been shown to activate the RAAS and the sympathetic nervous system, to elevate ET-1 and ROS and to decrease NO, which all predisposes to hypertension.^188^ Also, these drugs frequently cause nephrotoxicity that could lead to renal sodium retention, further contributing to prohypertensive effects.^188^

Radiotherapy and/or extensive surgery of the head and neck can provoke hypertension and even hypertensive crisis as a consequence of carotid baroreflex failure, leading to increased sympathetic activity and an increase in ROS.^189,190^ Additionally, abdominal radiotherapy has been associated with renal arterial stenosis which may occasionally provoke severe renovascular hypertension.^191^ Via the promotion of atherosclerosis and vasculopathy, radiotherapy may further elevate cardiovascular risk. Indeed, there is a notable increase in the incidence of cardiovascular and cerebrovascular events in patients who have undergone radiation therapy, particularly of the thorax, head, or neck.^192^

## Management of Hypertension Related to Anticancer Drugs

**Figure 4.**
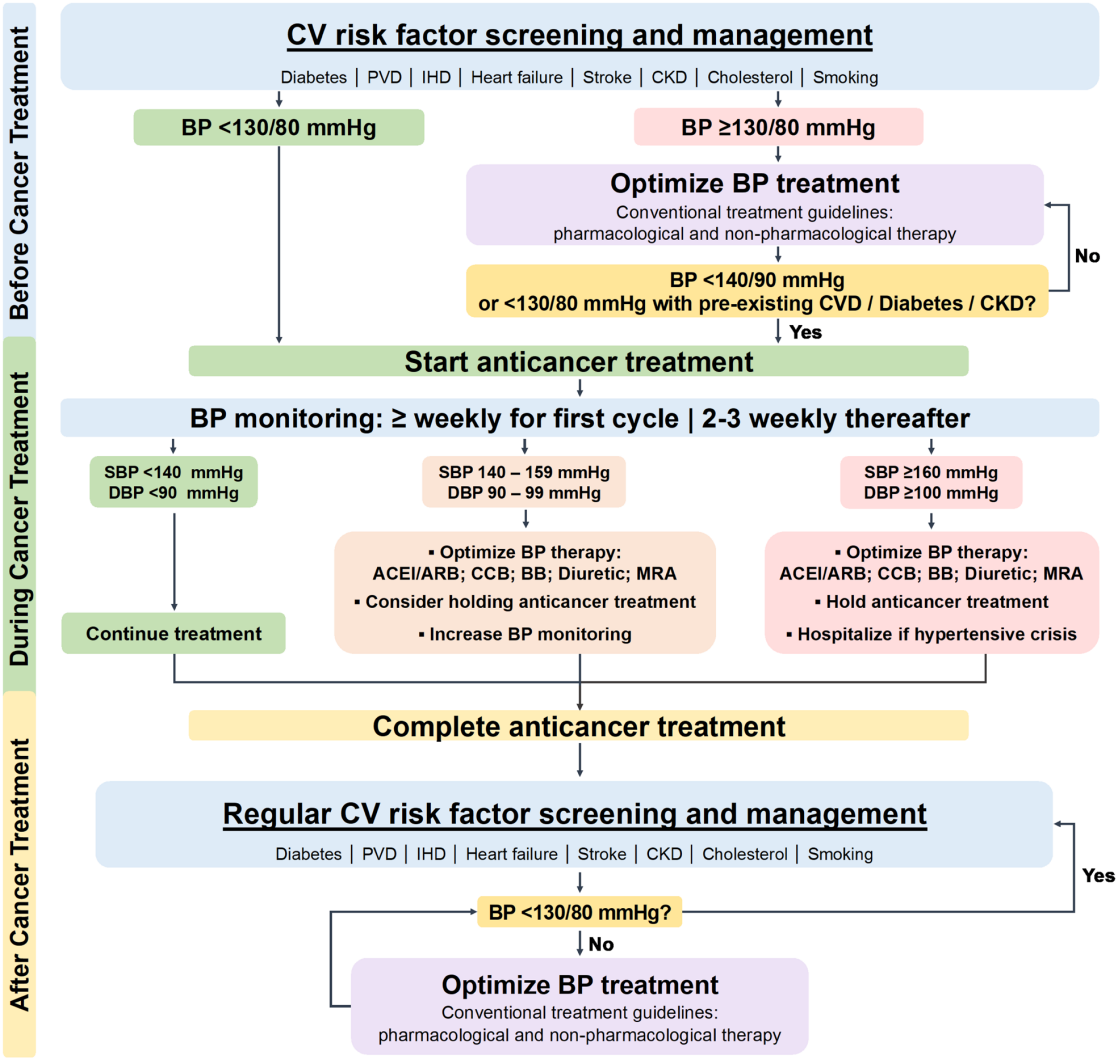
**Algorithm for the screening, monitoring, and treatment of blood pressure in patients with cancer receiving antineoplastic therapy known to be associated with hypertension.** ACEI indicates angiotensin-converting enzyme inhibitor; ARB, angiotensin II receptor blocker; BB, β-blocker; BP, blood pressure; CCB, dihydropyridine calcium channel blocker; CKD, chronic kidney disease; CVD, cardiovascular disease; DBP, diastolic blood pressure; IHD, ischemic heart disease; MRA, mineralocorticoid receptor antagonist; PVD, peripheral vascular disease; and SBP, systolic blood pressure.

The increasing number of patients with cancer presenting with CVD and particularly hypertension has stimulated the development of several cardio-oncology position statements and guidelines.^5,193–195^ These highlight the importance of a multidisciplinary input from cardiology, (hemato-)oncology, and cardiovascular specialists in the monitoring and management of CVD in this population, preferably in designated cardio-oncology teams. Nonetheless, the lack of evidence to support this guidance means there is inconsistency in the recommendations, and optimal management of CVD and hypertension in these patients can pose a major challenge for clinicians. In the following section, recommendations for the monitoring and management of hypertension before, during and after anticancer therapy are provided, which are summarized in Figure 4.

### Before Cancer Treatment

#### Cardiovascular Risk Stratification and Screening

As hypertension is the most prevalent comorbidity in patients diagnosed with cancer,^26^ the management and monitoring of hypertension begins before commencing anticancer treatment. This includes a detailed clinical history focused on cardiovascular risk factors, including hypertension, diabetes, and renal disease. Particular attention should be paid to a history of CVD, such as ischemic heart disease, cerebrovascular disease, peripheral arterial disease, and heart failure. A physical examination and focused investigations to screen for cardiovascular risk factors and end-organ damage should be performed.^63,66,106^ Where possible, ambulatory blood pressure monitoring or home blood pressure monitoring should be used to identify preexisting hypertension, and office blood pressure should always be measured before commencing treatment.^61,196^ Standard laboratory determinations, including total cholesterol, triglycerides, fasting plasma glucose, and renal function should be assessed at baseline. When anticancer agents with cardiotoxic potential are to be administered, an electrocardiogram and echocardiogram should be performed at baseline.

It is important to achieve optimal blood pressure control before commencing antineoplastic therapy, particularly in patients due to be exposed to agents known to have a pro-hypertensive profile and especially in those with baseline cardiovascular risk factors. It is particularly important that these management decisions are made collaboratively and proactively, preferably in a multidisciplinary cardio-oncology team, with the aim of achieving a balanced approach to minimize or avoid any potential delay in starting what may be urgent anticancer therapy. The aim should be to reduce the risks of adverse hypertension-induced end-organ effects, and to reduce the need for subsequent anticancer therapy interruption or dose reduction because of incident hypertension.

Given that hypertension is an independent predictor of cardiac events in cancer patients^16,60^ and that numerous anticancer agents exert prohypertensive effects, there is clear clinical value in developing risk-stratification tools that are validated in patients with cancer. These could aid in the identification of those at greatest risk for the development of treatment-related hypertension and, in particular, hypertension-related end-organ complications. Although risk stratification tools for the development of cardiotoxicity due to antineoplastic therapy have been developed,^197^ specific risk stratification tools for hypertension are lacking. Therefore, clinical assessment should focus on conventional cardiovascular risk factors. Particular attention should be paid to the identification and treatment of hypertension when therapies with potential cardiovascular toxicity are to be given in combination.

#### Blood Pressure Measurement and Monitoring

Blood pressure measurement in the clinic should be performed according to clinical guidelines to obtain reliable values.^198–200^ If possible, home blood pressure monitoring or ambulatory blood pressure monitoring should be used to monitor blood pressure before and during treatment, particularly in patients receiving or due to receive antineoplastic therapy that is associated with prohypertensive effects.^201^ Special attention should be given to adequate control of pain and anxiety in cancer patients, as these factors can directly lead to an elevation of blood pressure.^65^

The criteria for the diagnosis of hypertension varies between international guidelines, largely as a result of different interpretations of data from the SPRINT (Systolic Blood Pressure Intervention Trial).^202^ This trial demonstrated that cardiovascular outcomes in high risk patients were improved with intensive blood pressure control.^202^ The American College of Cardiology/American Heart Association guidelines recommend more stringent blood pressure control, with antihypertensive therapy to be commenced in patients when blood pressure is >130/80 mm Hg in the context of cardiovascular risk (defined as preexisting CVD, a calculated 10-year cardiovascular risk of >10%, or those with other cardiovascular risk factors such as kidney disease or diabetes).^198^ The current European Society of Cardiology guidelines and the European Society of Cardiology position paper on anticancer therapy cardiovascular toxicity recommend a treatment threshold of >140/90 mm Hg.^5,200^ Importantly, the trials on which these recommendations were based have not been conducted in cancer patient populations and extrapolation of these recommendations requires careful consideration. Clearly, blood pressure targets might be more lenient for cancer patients in the palliative setting, for whom the short-term benefits of anticancer therapy upon their quality of life might outweigh the increased risk of developing CVD in the long-term. In this population, adequate monitoring of acute hypertension-related effects may be most important.

We generally recommend a target blood pressure of <130/80 mm Hg before starting anticancer treatment, taking these recommendations and the increased risk of hypertension associated with some anticancer therapies into consideration. While initiation of anticancer treatment should not be delayed to achieve strict blood pressure control (these may be achieved in parallel), blood pressure should be at least <140/90 mm Hg before starting anticancer treatments with prohypertensive effects, in line with the National Cancer Institute Investigational Drug Steering Committee’s recommendations for initiating VEGFI therapy.^196^ In patients with preexisting CVD, diabetes, or proteinuric kidney disease, blood pressure control should be stricter (<130/80 mm Hg) before starting anticancer therapies associated with prohypertensive effects. The decision-making process on antihypertensive therapy, blood pressure targets, and timing of anticancer therapy should involve input from all members of the cardio-oncology team to ensure optimal cardiovascular status is achieved before treatment.

### During Cancer Treatment

Regular monitoring of blood pressure throughout cancer treatment is strongly advised. This is particularly relevant in the period soon after the initiation of anticancer therapy to detect acute rises in blood pressure.^61^ Therefore, we recommend that blood pressure is measured twice daily via home blood pressure monitoring during the first treatment cycle or first period of treatment. Home blood pressure monitoring may not be appropriate in all patients^203^ and in this setting, blood pressure measurements via the primary care physician at least once a week might be most suitable and these patients should be assessed on a case-by-case basis. If blood pressure levels remain within normal limits, the frequency of monitoring could be decreased to once every 2 to 3 weeks throughout treatment.^196^

#### Diagnosis and Management of Hypertension

While we recommend a target blood pressure <130/80 mm Hg before anticancer therapy, we suggest that during cancer treatment, antihypertensive therapy should only be commenced in patients with new onset hypertension whose blood pressure exceeds 140/90 mm Hg. In patients with preexisting CVD, diabetes or proteinuria, blood pressure treatment should be started if values exceed 130/80 mm Hg. This is suggested to reduce the risk of iatrogenic hypotension and to reduce the potential of inappropriate interruption of anticancer therapy.

Antihypertensive treatment may also be considered in patients who do not meet these definitions, but who have a substantial acute rise in blood pressure (eg, SBP rise >20 mmHg) after initiation of anticancer therapy. It is unclear whether absolute blood pressure or the magnitude of change in blood pressure from baseline is a more important contributor to end-organ dysfunction, but it might be that particularly this rapid increase in blood pressure predisposes patients to hypertensive complications.^13,14^ Furthermore, a previous meta-analysis demonstrated that every 10 mmHg reduction in SBP significantly reduces the risk of major cardiovascular events, such as coronary heart disease (RR, 0.8), stroke (RR, 0.73), and heart failure (RR, 0.72).^204^ It is unknown whether the converse holds true, and a similar size of increased cardiovascular risk is associated with blood pressure elevations of the same magnitude. This may be of particular relevance in patients receiving potentially prohypertensive anticancer therapies. This important gap in knowledge would aid in the recommendation of blood pressure treatment targets.

Currently, specific guidance on the choice of antihypertensive agents in the cancer population is lacking. When choosing antihypertensive agents, it is important that careful consideration is given to drug pharmacokinetics and pharmacodynamics, as well as the presence of comorbidities and potential side effects. For example, nondihydropyridine calcium channel blockers (CCB), such as verapamil and diltiazem, should not be used in combination with most VEGFI as they have the potential for cytochrome P450 inhibition, which may lead to elevated circulating VEGFI concentrations.^5,62^ The choice of first-line antihypertensive agent generally follows national guidelines for hypertension management, as there is currently no clinical evidence to support the use of one class of agent over another.^5,205,206^

Dihydropyridine CCB such as amlodipine or nifedipine potently reduce arterial smooth muscle cell contractility in blood vessels.^207^ Therefore, given the role vascular dysfunction plays in the development of hypertension with many anticancer agents (Figure 3), CCB are likely to be effective in this setting. Indeed, a study of 36 patients with nonsmall cell lung cancer, ovarian cancer and colorectal cancer who developed hypertension with bevacizumab therapy demonstrated the efficacy of amlodipine in achieving blood pressure control.^208^ Nonetheless, it is often necessary to combine 2 or more antihypertensive agents to achieve satisfactory blood pressure levels.^206^ In this setting, combination of an ACEI or an angiotensin II receptor blocker (ARB) with a CCB is generally accepted as the first choice (Figure 4).^5,198,205^

Despite a lack of robust evidence for a major role of the RAAS in the prohypertensive mechanisms of most anticancer therapies, ACEI and ARB are frequently used in the treatment of VEGFI-associated hypertension and in the treatment of hypertension associated with other cancer therapies. ACEI/ARB may be used preferentially in cancer patients with diabetic nephropathy, left ventricular systolic dysfunction (LVSD), and cancer-therapy associated proteinuria. Other antihypertensive agents such as diuretics and β-blockers may be used when ACEI/ARB and CCB are ineffective in normalizing blood pressure.^209^ Of note, diuretics should be prescribed with caution for patients at risk of dehydration. Mineralocorticoid receptor antagonists such as eplerenone and spironolactone are increasingly being administered to patients with resistant hypertension,^210^ and they may be a useful option in the management of cancer therapy-associated hypertension that is unresponsive to conventional therapy.

Any decision about altering the dose or withholding anticancer therapy should be made via a multidisciplinary approach involving input from all members of the cardio-oncology team. Generally, in patients who are symptomatic or have new evidence of end organ damage, dose reduction or temporary withdrawal of anticancer therapy should be considered. In cases of severe hypertension (≥160/100 mm Hg), temporary withdrawal of anticancer therapy until adequate blood pressure control is achieved is recommended (Figure 4). Notably, careful follow-up of the efficacy and tolerability of antihypertensive drugs is warranted to monitor the occurrence of rebound hypotension, particularly during off-treatment periods or after definite termination of the anticancer therapy.

### After Cancer Treatment

As cancer treatments continue to improve cancer prognosis, there is a growing clinical need for robust guidelines for long-term monitoring and management of CVD and risk factors in cancer survivors. However, current recommendations mainly focus on cardiovascular screening and monitoring of cardiac function before or during treatment.^194,211^ To date, the most comprehensive recommendations for the monitoring of cardiovascular risk factors and CVD have been focused on survivors of hematologic malignancies who have undergone stem cell transplantation.^212,213^ These guidelines recommend regular screening for cardiovascular risk factors following the completion of anticancer therapy, including obesity, hypertension, and diabetes. Guidelines that are applicable to the general cancer survivor population are lacking. We suggest that cancer survivors should have hypertension managed strictly and similarly to individuals with other cardiovascular risk factors. Therefore, we endorse a diagnostic and treatment blood pressure threshold of ≥130/80 mm Hg for all patients who have received prior anticancer drug therapy.

## Conclusions

The development of novel anticancer therapies has substantially improved the prognosis for patients with a wide variety of malignancies. Despite these favorable outcomes, many of these drugs induce a systemic hypertensive response during therapy that can limit the safe delivery of anticancer treatment. Furthermore, the rapidly growing number of cancer survivors are at increased risk from end-organ complications of hypertension. While there are shared risk factors and overlapping pathophysiological mechanisms underlying both cancer and hypertension, the precise mechanisms underlying prohypertensive effects of novel classes of antineoplastic agents remain incompletely defined. Careful assessment of blood pressure, cardiovascular risk factors, and potential end-organ effects is essential before, during, and after anticancer treatment. Currently, specific guidelines for screening, monitoring, and treatment of hypertension in the general oncological population are lacking but highly warranted. A collaborative approach between cardiologists, (hemato)-oncologists, and cardiovascular specialists remains vital in the day-to-day management of patients with cancer and hypertension. This team-based approach, including basic scientists, remains fundamental for the design of appropriate preclinical studies and clinical trials for future directions (Table 2) to better guide these complex intertwined issues. Only by doing so, will the unprecedented anticancer effects of novel and conventional agents be maximized while simultaneously minimizing cardiovascular risk.

**Table 2.**
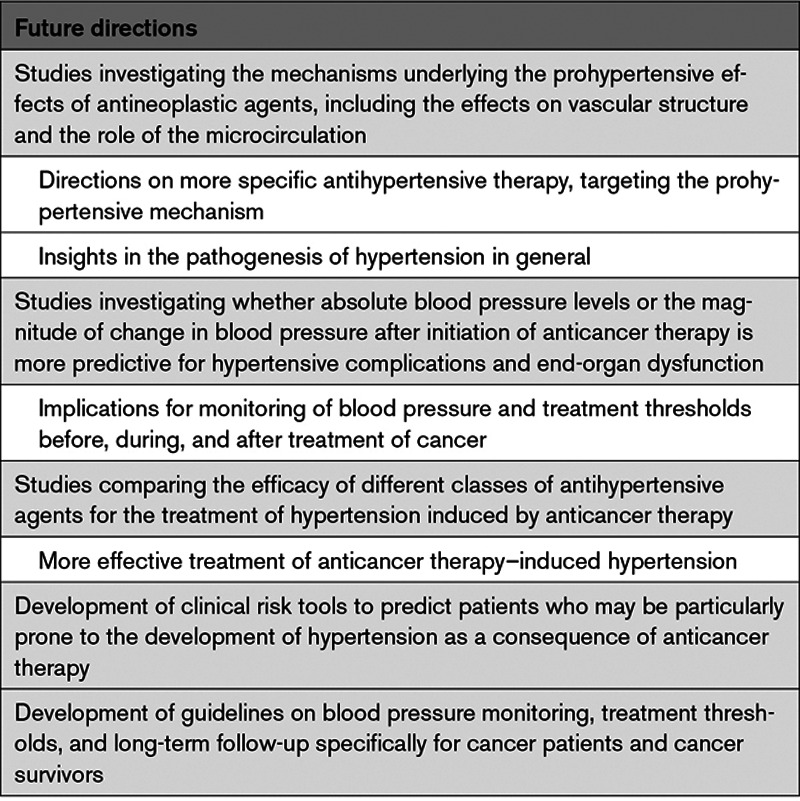
Future Directions

## Sources of Funding

S.J.H. Dobbin, K.B. Neves, and N.N. Lang are supported by a British Heart Foundation (BHF) Centre of Research Excellence grant (RE/18/6/34217) and a BHF Project Grant (PG/19/64/34434). S.M. Hermann is supported by National Institutes of Health K08 DK118120 from the NIDDK.

## Disclosures

N.N. Lang has received speaker’s fees from Roche, Pfizer, and Novartis and research grant support from Roche Diagnostics. The other authors report no conflicts.
